# Phytic Acid Treatment Inhibits Browning and Lignification to Promote the Quality of Fresh-Cut Apples during Storage

**DOI:** 10.3390/foods11101470

**Published:** 2022-05-18

**Authors:** Ting Fang, Jia Yao, Yuquan Duan, Yaoguang Zhong, Yaoyao Zhao, Qiong Lin

**Affiliations:** 1 College of Food Science and Technology, Shanghai Ocean University, Shanghai 201306, China; 18846094195@163.com; 2 Key Laboratory of Agro-Products Quality and Safety Control in Storage and Transport Process, Ministry of Agriculture and Rural Affairs/Institute of Food Science and Technology, Chinese Academy of Agricultural Sciences, Beijing 100193, China; duanyuquan@caas.cn (Y.D.); sdzyaoyao@126.com (Y.Z.); 3 School of Biomedicine, Beijing City University, Beijing 100094, China; yaojia866@126.com

**Keywords:** apple, phytic acid, fresh-cut, browning, lignification

## Abstract

Browning and lignification often occur in fresh-cut apple processing, leading to quality deterioration and limiting the shelf life of products. In this study, 0.8% (*v*/*v*) phytic acid was used to improve the quality and shelf life of fresh-cut apples. From the results, the browning was inhibited by the phytic acid treatment and the browning index (BI) of the control fruit was 1.62 times that of phytic acid treatment at 2 d of storage. The lignin content in phytic acid-treated fruit significantly decreased at 2, 4, and 6 d of storage compared to the control. Phytic acid treatment also reduced H_2_O_2_ and malonaldehyde (MDA) contents, which may indicate lighter membrane damage to apples. Compared with the control, the polyphenol oxidase (PPO) and peroxidase (POD) activities decreased while superoxide dismutase (SOD) and catalase (CAT) activities increased in phytic acid-treated fruit. Consistent with the lignin content, the activities of phenylpropane metabolism-related enzymes phenylalanine ammonia-lyase (PAL), cinnamate 4-hydroxylase (C4H), and 4-coumarate: CoA ligase (4CL) were inhibited by phytic acid treatment. In conclusion, phytic acid alleviated the browning and lignification of fresh-cut apples by reducing PPO and POD activities, maintaining cell membrane integrity, and inhibiting phenylpropane metabolism.

## 1. Introduction

Apple is widely produced and consumed in many countries and contains abundant nutrients, dietary fibers, and bioactive metabolites [1,2]. Fresh-cut apples are prepared for ready-to-eat food, packaged, and stored at low temperatures, thus providing convenience for consumers. However, the browning and lignification that occurred during fresh-cut processing greatly shortened the shelf life of the products. Previous reports indicated that the browning of fresh-cut apples mainly resulted from the oxidation of phenols catalyzed by polyphenol oxidase (PPO) and peroxidase (POD) [3,4]. Moreover, the lignin was found to accumulate in the wound area of fresh-cut apples [5], which led to the lignification of apple slices. Phenylpropane metabolism is involved in the formation of lignin. In phenylpropane metabolism, the phenylalanine ammonia-lyase (PAL) can catalyze phenylalanine deamination to generate trans-cinnamic acid [6]. The cinnamate 4-hydroxylase (C4H) and 4-coumarate: CoA ligase (4CL) respectively catalyze the conversion of trans-cinnamic acid and the synthesis of p-coumarate coenzyme-A to provide precursor substances for lignin synthesis [7,8]. In addition, fresh-cut apples exited other quality problems such as water loss and softening. Thus, it is a challenge to improve the preservation technologies for the fresh-cut apple industry.

Many technologies such as cold storage, aqueous ozone treatment [9], UV-C treatment [10], atmospheric gas plasma treatment [11], sulfites treatment, ascorbic acid treatment [12], γ-aminobutyric acid treatment [4], thyme oil-alginate-based coating [13], and lysozyme treatment [14] have been used on fresh-cut apples to prolong shelf life. However, some of them need specific devices and may cause allergic reactions if people use them frequently [15,16,17]. Therefore, there is a need to find an alternative method for preserving fresh-cut apples.

Phytic acid is a highly phosphorylated molecule abundant in plants [18], and is generally recognized as safe according to U.S. Food and Drug Administration [19]. It has been used in the food industry to reduce the browning of fruit and vegetables by inhibiting the activities of POD and PPO enzymes [20]. The litchi pulp quality and pericarp color were maintained intact, and the preservative life was up to 40 d by phytic acid treatment [21]. Phytic acid, as a natural antioxidant, can restrain lipid peroxidation by decreasing the accumulation of iron-catalyzed hydroxyl radical [22]. It can improve the oxidative stability in both raw and cooked meat at refrigerated temperatures [23]. In addition, the composite preservative of phytic acid and ascorbic acid could reduce weight loss rate, cell permeability, and PPO activity and maintain the sensory quality of mushrooms [24]. For now, phytic acid has been reported to maintain moisture and chlorophyll content in fresh-cut red cabbage [25]. However, there is little research on the phytic acid application in fresh-cut apples, and its preservation mechanism is unclear.

This study aims to investigate the storage quality and preservation mechanism of fresh-cut apples by phytic acid treatment. The parameters including color, total phenol content, lignin content, membrane damage, and related enzyme activities were measured. This work will provide a choice for the food industry on fresh-cut apple preservation.

## 2. Materials and Methods

### 2.1. Sample Preparation and Treatments

Fresh ‘Fuji’ apple (*Malus domestica*) was bought from the Xinfadi market in Beijing, China, and stored at 4 °C before the experiments. Fruits of uniform size and color without the physiological disorder, infection, or mechanical damage were selected. A total of 80 apples were rinsed gently by hand, using distilled water, and were dried at room temperature. Then apples were peeled, cored, and cut into 12 pieces with an apple slicer. All the slices were soaked in 5 g L^−1^ sodium chloride (Sinopharm Chemical Reagent Co. Ltd., Beijing, China) solution to inhibit browning before phytic acid treatment. The slices were used for the following treatments: the control (soaked in distilled water) and 0.8% (*v*/*v*) phytic acid (Shanghai Source Leaf Biotechnology Co., Ltd., Shanghai, China) solution treatments. Soak time was 10 min, then they were drained with blotting paper. After treatments, samples in two groups were packaged in plastic boxes with lids and the headspace volume was about 20% of the volume of the box. Then, samples were stored at 4 °C and taken at 0, 2, 4, 6, and 8 d of storage. The apple slices were cut into small cubes, frozen immediately with liquid nitrogen, and stored at −80 °C for further analysis.

### 2.2. Sensory Evaluation

Sensory evaluation was performed at 0, 2, 4, 6, and 8 d of storage according to Table 1 [26,27]. A panel of 8 assessors (4 males and 4 females, 25~40 years old) was selected and trained. Five key attributes, including color, smell, texture, taste, and acceptability, were selected for evaluation. A scale of 0 to 20 was used for each attribute in the sensory evaluation. The sensory score for each sample was calculated as the mean value. Fresh-cut apples with a sensory score below 60 are considered unacceptable.

### 2.3. Total Bacterial Count

The total bacterial count of fresh-cut apples was measured at 0, 2, 4, 6, and 8 d of storage and calculated according to the Chinese GB standard (GB4789.2-2016). The determination of total bacterial count was carried out in triplicate, and the result was expressed as log_10_ CFU g^−1^.

### 2.4. Color Parameters

The pictures of apple slices were taken at each sampling point. Flesh color parameters of fresh-cut apples were measured using a colorimeter (CS-200, Hangzhou Caipu Technology Co., Ltd., Hangzhou, China) equipped with an 8 mm diameter measuring area. Before measurement, the colorimeter was calibrated with a black and white calibration plate. The results were based on CIELAB (L*, a*, b*) color space. ‘L*’, ‘a*’, and ‘b*’ represent respectively dark-lightness, red-greenness, and blue-yellowness. ‘L*’, ‘a*’, and ‘b*’ values of apple flesh were measured on each sampling day. Each treatment had three replicates. Six apple pieces were measured for each replicate. The browning index (BI) indicating the intensity of brown color was calculated using Equation (1) according to Liu et al. [3]:

(1) $$\mathrm{BI}=\frac{100}{0.172}\times \left(\frac{a^{*}+1.75L^{*}}{5.645L^{*}+a^{*}-3.012b^{*}}-0.31\right)$$

### 2.5. Weight Loss Rate

Weight loss was assessed by the weight difference of six apple slices after storage using an electronic balance (YP6001, Shanghai Youke Instrument Co., Ltd., Shanghai, China) with an accuracy of 0.1 g, and measured every 2 days. Each treatment had three replicates. ‘m_0_’ represents the weight at 0 d, and ‘m_1_’represents the weight at each sampling day. The weight loss rate was calculated through Equation (2) according to Chen et al. [28]:

(2) $$\mathrm{Weight}\;\mathrm{loss}\;\mathrm{rate}=\frac{m_{0}-m_{1}}{m_{0}}\times 100\%$$

### 2.6. Total Phenol Content and Lignin Content

The total phenol content was measured according to Ge et al. [29] with some modifications. 1 g of frozen powder was homogenized with 5 mL pre-cooled 1% (*v*/*v*) HCl-methanol, then centrifuged at 12,000× *g* at 4 °C for 20 min. The supernatant was collected and placed on ice for measurement. The 1% HCl-methanol solution was used as a blank reference to zero, and the absorbance value of the supernatant was measured by a spectrophotometer (T6 new century, Beijing Pgeneral Instrument Co., Ltd., Beijing, China) at 280 nm wavelength, repeated three times. The total phenol content was expressed as optical density (OD)_280_ kg^−1^.

The lignin content was detected by BC4205 kit (Beijing Solarbio Science and Technology Co., Ltd., Beijing, China) following the manufacturer’s instructions. Perchloric acid (Sinopharm Chemical Reagent Co. Ltd., Beijing, China) and glacial acetic acid (Fuchen (Tianjin) Chemical Reagent Co., Ltd., Tianjin, China) were used in this method. The lignin content was measured at 280 nm by a microplate reader (Spark, Tecan Group Ltd., Männedorf, Switzerland) and expressed as a percentage.

### 2.7. H_2_O_2_ and Malonaldehyde (MDA) Contents

The H_2_O_2_ and MDA contents were detected by BC0025 and BC3595 kits respectively (Beijing Solarbio Science and Technology Co., Ltd., Beijing, China) following the manufacturer’s instructions. Acetone (Sinopharm Chemical Reagent Co. Ltd., Beijing, China) and chlorhydric acid (Beijing Chemical Works Co., Ltd., Beijing, China) were used to measure H_2_O_2_. The H_2_O_2_ content was measured at 415 nm by a microplate reader and expressed as mmol kg^−1^. The MDA content was measured at 450, 532, and 600 nm by a microplate reader and expressed as μmol kg^−1^.

### 2.8. Enzyme Activity

The PPO and POD activities were detected using G0113W and G0107W kits respectively (Suzhou Grace Biotechnology Co., Ltd., Suzhou, China). PAL, superoxide dismutase (SOD), and catalase (CAT) activities were detected using BC0215, BC0175, and BC0200 kits respectively (Beijing Solarbio Science and Technology Co., Ltd., Beijing, China). C4H and 4CL activities were detected using TE0407 and TE0411 kits, respectively (Beijing Leagene Biotechnology Co., Ltd., Beijing, China). The measuring steps were performed following the manufacturer’s instructions. The PPO, POD, PAL, SOD, CAT, C4H, and 4CL activities were respectively measured at 420, 470, 290, 560, 240, 290, and 333 nm by a microplate reader. One unit of SOD activity was defined as the amount of enzyme that caused 50% inhibition of nitro blue tetrazolium reduction. One unit of CAT activity was defined as the amount of enzyme that decomposed 1 μmol H_2_O_2_ per minute. One unit of PPO, POD, PAL, and 4CL activities was respectively defined as a change of 0.01, 1, 0.1, and 0.01 units in absorbance value per minute. One unit of C4H activity was defined as a change of 0.01 units in absorbance value per hour. The activities of these enzymes were expressed as U kg^−1^.

### 2.9. Statistical Analysis

The determination of each indicator was repeated at least three times. The data were analyzed via one-way analysis of variance (ANOVA) in Microsoft Office Excel 2019 (Microsoft, Redmond, WA, USA) and SPSS Statistics 20 (IBM, Armonk, NY, USA). Duncan’s test at the 0.05 level was used to compare the mean averages. Origin Pro 8.6 (Microcal Software, Northampton, MA, USA) was used for graph drawing.

## 3. Results

### 3.1. Sensory Evaluation

**Figure 1:**
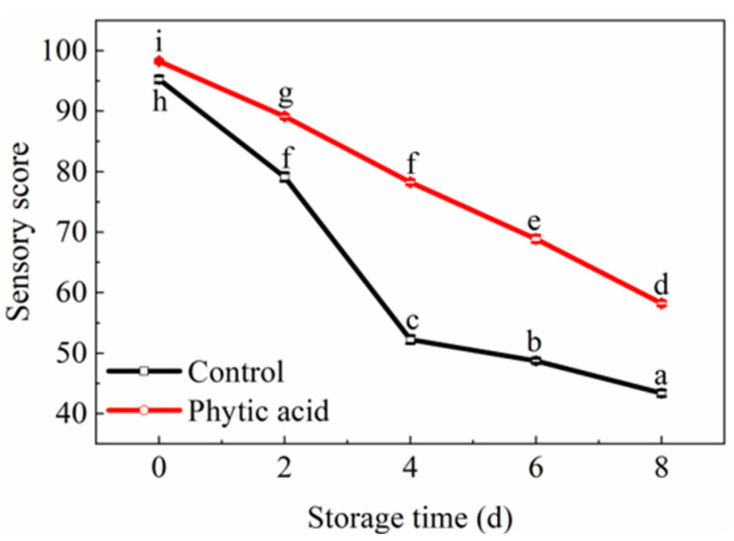
Sensory evaluation of fresh-cut apples during storage after treatment with phytic acid or the control. Vertical bars represent standard errors of the means of eight replicates. The different letters indicate significant differences at *p* < 0.05.

As shown in Figure 1, the sensory scores of fresh-cut apples showed a downward trend in the control and phytic acid groups during storage. On the first day, it was lower in the control than that in phytic acid treatment due to rapid browning. Moreover, the sensory score of phytic acid treatment was significantly higher than that of the control during storage. At 4 d of storage, the sensory score of fruit in the control was lower than 60, while that in the phytic acid was close to 80. The above results suggested that phytic acid can prevent the deterioration of the sensory quality in fresh-cut apples.

### 3.2. Total Bacterial Count

The total bacterial counts of fresh-cut apples increased gradually during the whole storage period, and there was no significant difference between the control and the phytic acid-treated apples (Supplementary Figure S1). For safe consumption, the Spanish legal limit (RD 3484/2000, 2001) for microbial populations on fresh-cut fruit is 7 log_10_ CFU g^−1^. In our study, the total bacterial counts of fresh-cut apples in two treatments at 8 d of storage were lower than 7 log_10_ CFU g^−1^.

### 3.3. Flesh Color

**Figure 2:**
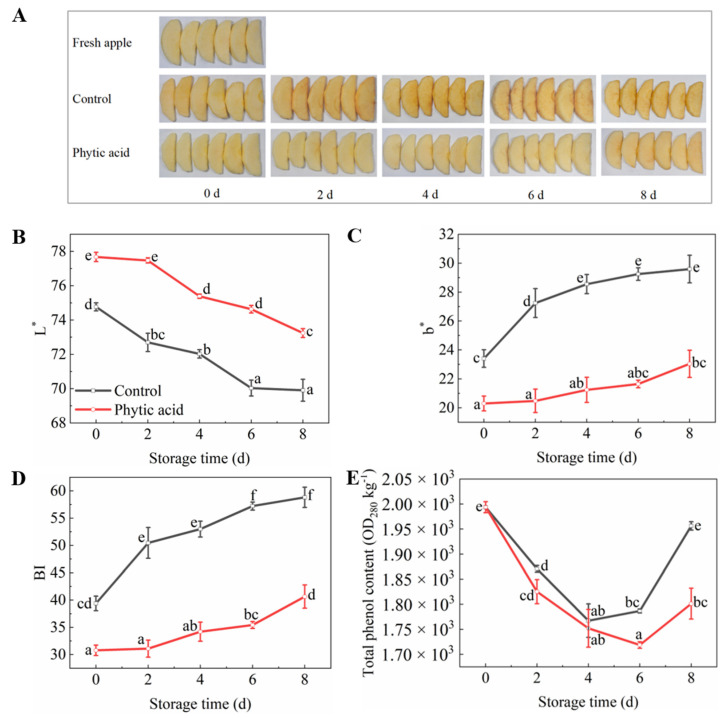
Photos and color parameters of fresh-cut apples during storage after treatment with phytic acid or the control. (**A**) Photos; (**B**) L*; (**C**) b*; (**D**) BI; (**E**) Total phenol content. The ‘Fresh apple’ represents unsoaked apple slices. Vertical bars represent standard errors of the means of three replicates. The different letters indicate significant differences at *p* < 0.05.

Phytic acid-treated fruits maintained their original flesh color at 4 d of storage, greatly prolonging their shelf life. However, the apples in the control turned brown at 0 d of storage (Figure 2A). Consistent with the pictures, phytic acid treatment can significantly restrain the changes in color parameters. The ‘L*’ value was higher while the ‘b*’ value was lower in phytic acid-treated apples than those in the control (Figure 2B,C). The BI value of the control fruit was significantly higher compared to the phytic acid-treated apples. At 2 d of storage, the BI value of the control fruit was 1.62 times that of phytic acid treatment (Figure 2D). The total phenol content of apples decreased in both treatments at the beginning of storage, however, phytic acid treatment delayed the increase of total phenol content in the late storage period (Figure 2E). These results indicated that phytic acid may inhibit browning by decreasing phenol content accumulation in fresh-cut apples.

### 3.4. Flesh Lignification

**Figure 3:**
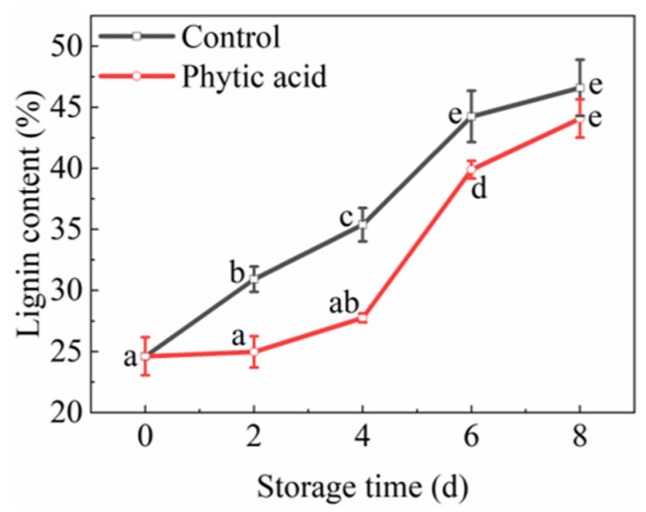
Lignification of fresh-cut apples during storage after treatment with phytic acid or the control. Vertical bars represent standard errors of the means of three replicates. The different letters indicate significant differences at *p* < 0.05.

The weight loss rate of fresh-cut apples increased gradually in phytic acid treatment and the control during storage, but no significant difference was observed between the two treatments (Supplementary Figure S2). In both phytic acid treatment and the control, the lignin content of apples showed an increasing trend during storage. The lignin contents in phytic acid-treated fruit were significantly decreased at 2, 4, and 6 d of storage compared to the control (Figure 3). At 4 d of storage, the lignin contents in phytic acid-treated fruit and the control were 27.75 and 35.38%, respectively. Thus, phytic acid delayed the lignification of apple slices.

### 3.5. H_2_O_2_ and MDA Contents

**Figure 4:**
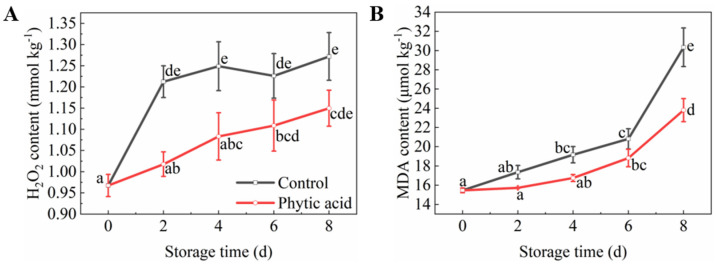
Membrane damage indicators of fresh-cut apples during storage after treatment with phytic acid or the control. (**A**) H_2_O_2_ content; (**B**) MDA content. Vertical bars represent standard errors of the means of three replicates. The different letters indicate significant differences at *p* < 0.05.

H_2_O_2_ and MDA are associated with membrane damage. The accumulation of H_2_O_2_ in fresh-cut apples gradually increased during storage, however, phytic acid treatment reduced its content during storage compared with that in the control (Figure 4A). The H_2_O_2_ content of the control fruit before 4 d of storage was significantly higher than that in phytic acid treatment. The MDA content in both treatments increased during the whole storage period and phytic acid treatment inhibited the accumulation of MDA (Figure 4B). These results implied that phytic acid treatment was conducive to reducing membrane damage of apple slices.

### 3.6. Browning-Related Enzymes Activities

**Figure 5:**
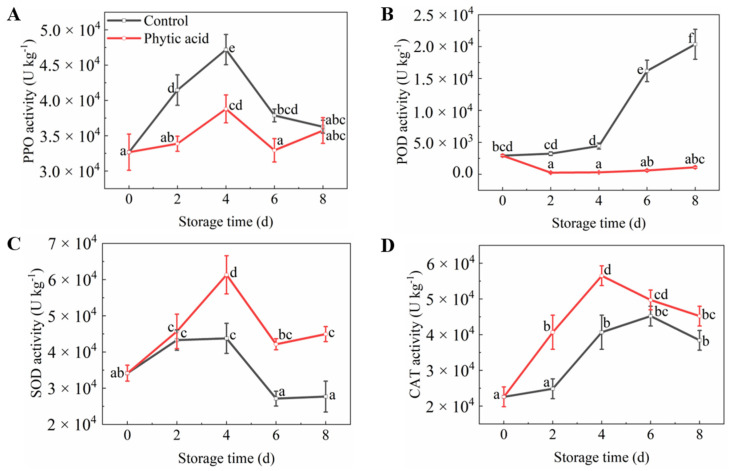
Browning-related enzyme activities of fresh-cut apples during storage after treatment with phytic acid or the control. (**A**) PPO activity; (**B**) POD activity; (**C**) SOD activity; (**D**) CAT activity. Vertical bars represent standard errors of the means of three replicates. The different letters indicate significant differences at *p* < 0.05.

PPO activity first increased and then decreased at 6 d of storage in both treatments, and phytic acid suppressed its activity compared to that in the control (Figure 5A). Meanwhile, the POD activity of apple slices showed an increasing trend in the control while being maintained at a low level in phytic acid-treated fruits. Moreover, it was significantly lower in phytic acid-treated apple slices during the whole storage period compared to the control (Figure 5B). The SOD activity of fruit increased at the beginning of storage and declined at 4 d of storage in both treatments. Compared to the control, the SOD activity of fresh-cut apples treated with phytic acid was enhanced during storage. And its activity in treatment was 1.55 times that of the control at 6 d of storage (Figure 5C). In addition, phytic acid treatment increased the CAT activity of fresh-cut apples during storage (Figure 5D). The above results indicated that phytic acid inhibited browning in apple slices.

### 3.7. Phenylpropane Metabolism-Related Enzymes Activities

**Figure 6:**
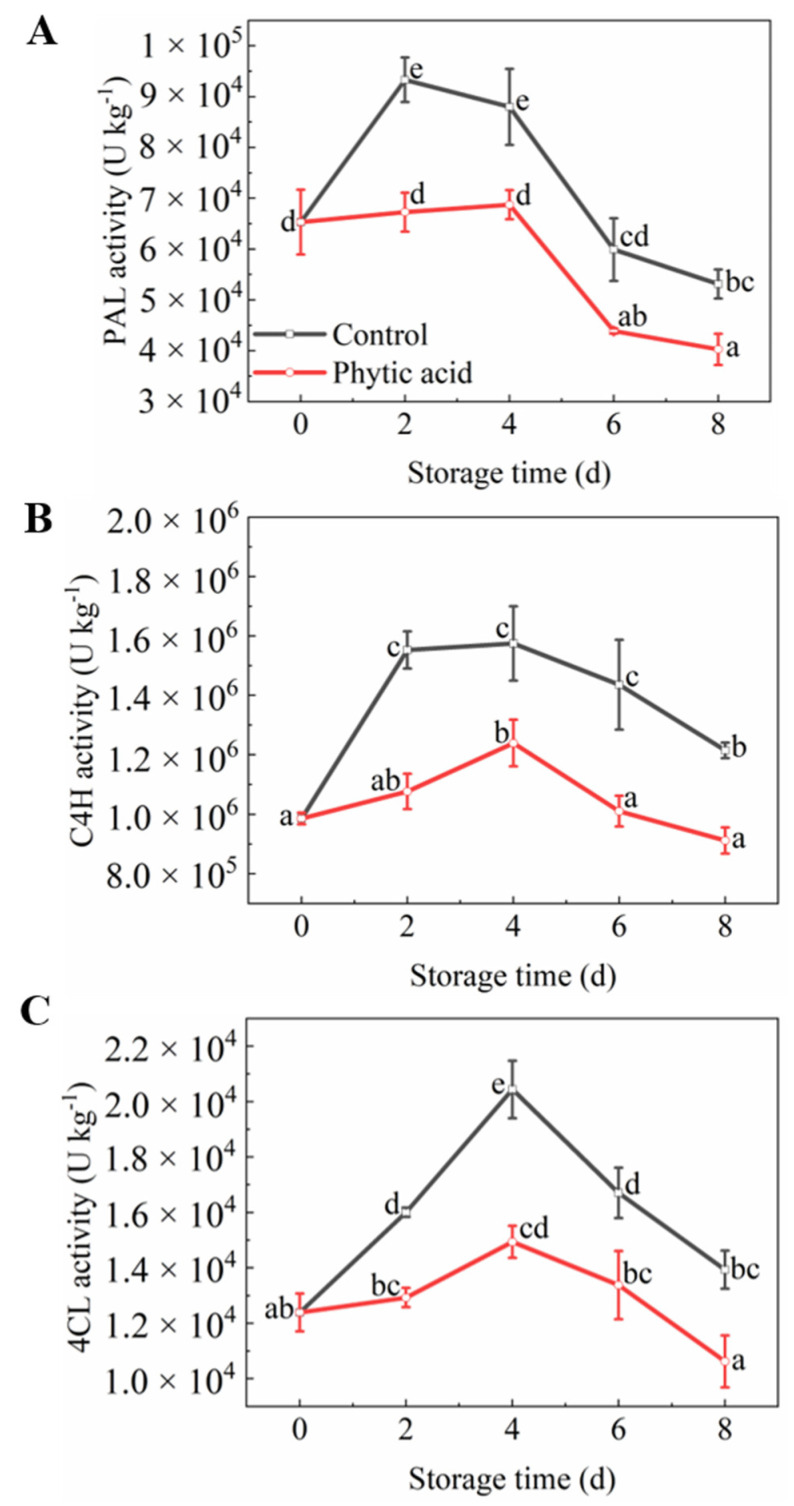
Phenylpropane metabolism-related enzyme activities of fresh-cut apples during storage after treatment with phytic acid or the control. (**A**) PAL activity; (**B**) C4H activity; (**C**) 4CL activity. Vertical bars represent standard errors of the means of three replicates. The different letters indicate significant differences at *p* < 0.05.

The activities of three phenylpropane metabolism-related enzymes, PAL, C4H, and 4CL, in fresh-cut apples, increased and then decreased during storage (Figure 6). The PAL activity significantly decreased after phytic acid treatment compared to the control during the whole storage period (Figure 6A). The C4H activity in phytic acid-treated apples fluctuated little and decreased compared with the control during the whole storage period (Figure 6B). 4CL activity increased to a high level in the control fruit while it was more stable in phytic acid treatment. Moreover, its activity in phytic acid-treated fruit was 73.1% of that in the control at 4 d of storage (Figure 6C). The above results showed that phytic acid restrained phenylpropane metabolism in apple slices.

## 4. Discussion

The acceptability of fresh-cut fruit and vegetables is related to their appearance and texture [30]. During processing and storage, apple flesh gradually turned brown, which greatly affected the sensory quality and storage time of fresh-cut apples. The decrease in the ‘L*’ value and increases in the ‘b*’ and BI values of apple flesh occur in the browning process [12]. Adding phytic acid to meats, aquatic products, fruit, vegetables, and other foods could maintain color and prolong preservation time [31]. From our results, the phytic acid-treated apple slices showed higher ‘L*’ values and lower ‘b*’ and BI values compared with the control, indicating phytic acid can inhibit the browning of fresh-cut apples during storage.

The browning of fresh-cut apples is mainly due to enzymatic browning. PPO and POD, two major enzymes of browning, catalyze the oxidation of phenols in plant tissues in the presence of oxygen, resulting in the accumulation of melanin, which is a brown or black pigment related to ‘browning’ [4,30,32]. From our results, the phytic acid could delay browning and inhibit PPO and POD activities, which was consistent with previous research [33,34]. In addition, phytic acid treatment reduced total phenol content, resulting in the alleviation of enzymatic browning. The total phenol content of fresh-cut apples decreased at the beginning of storage, which may be due to lower phenol synthesis rates than oxidation rates [28]. These results indicated phytic acid could suppress the browning of fresh-cut apples by reducing phenols content and restraining PPO and POD activities.

It is well known that membrane disruption due to cutting brings the enzymes into connection with their substrates, thus browning occurs [35]. In addition, many studies showed the accumulation of reactive oxygen species (ROS) resulted in the oxidation of the cell membrane and the damage to cell membrane structure [36,37]. In fresh-cut apples, the accumulation of H_2_O_2_ during cutting may stimulate membrane lipid peroxidation, destroy cellular membrane structure and produce MDA, which results in the browning of apple slices [38]. MDA has been reported as an indicator of cell membrane integrity [35]. In this study, the MDA content of the control fruits was higher than that of fruits in phytic acid treatment, indicating that phytic acid maintained the cell membrane structure of fresh-cut apples.

The antioxidant enzymes, SOD and CAT, are involved in alleviating cell membrane damage in plants. SOD catalyzes the dismutation of superoxide anion to H_2_O_2_, which can be decomposed by CAT, thus reducing ROS content and relieving membrane lipid peroxidation [6,38]. According to Mahunu et al. [39], the synergistic effect of phytic acid and yeast on CAT activity was regarded as the ability to scavenge excessive ROS and maintain low ROS concentration. Moreover, phytic acid supplementation provided higher levels of SOD and CAT activities in the diet [40]. Our results showed that phytic acid increased the activities of SOD and CAT, thus maintaining the cell membrane integrity of fresh-cut apples.

The mechanical injury during the processing of fresh-cut apples gives rise to the generation of a wound-healing response and the structural barrier such as lignin [41]. The phenylpropanoid pathway is an important step in the synthesis of lignin, causing the lignification of fresh-cut apples. PAL catalyzes the first step of phenylpropanoid metabolism, which is directly relevant to the synthesis of lignin [7]. High-pressure argon and xenon mixed treatment increased PAL activity and enhanced the accumulation of lignin in fresh-cut apples [5]. C4H involves in the second step of the phenylpropanoid pathway, catalyzing the conversion of cinnamic acid to coumaric acid, and 4CL regulates the synthesis of different types of lignin monomers [42]. Sodium nitroprusside effectively enhanced the activities of PAL, C4H, and 4CL, and increased the accumulation of lignin in apple fruit [43]. Zhou et al. [8] found ascorbic acid treatment inhibited the wound healing of fresh-cut potato strips by inhibiting PAL, C4H, 4CL, and POD activities. POD can catalyze the polymerization of lignin monomer in the last step of lignin biosynthesis [44]. From our results, it can be inferred that phytic acid reduced the accumulation of lignin and lignification by suppressing PAL, C4H, 4CL, and POD activities.

## 5. Conclusions

A phytic acid concentration of 0.8% was used to improve the quality and prolong the shelf life of fresh-cut apples. Phytic acid could alleviate the sensory quality deterioration, color changes, and lignin accumulation of apple slices during storage. Meanwhile, phytic acid was able to maintain cell membrane integrity by reducing the accumulation of H_2_O_2_ and MDA. In addition, PPO, POD, PAL, C4H, and 4CL activities were inhibited while SOD and CAT activities were increased by phytic acid treatment to inhibit the browning and lignification of apple slices. In general, the shelf life of apple slices in phytic acid treatment was extended to 4 days. It was concluded that phytic acid promoted the quality of fresh-cut apples by alleviating the browning and lignification during storage.

## Figures and Tables

**Table 1:** Indexes for the sensory evaluation in fresh-cut apples.

Item	Evaluation Index	Score
Color	The surface is clear and bright, uniform color.	≥14, <20
	The surface is slightly darker, with dark fibrous streaks.	≥8, <14
	The surface is dark, with brown spots.	<8
Texture	The surface does not shrink.	≥14, <20
	The surface slightly shrinks.	≥8, <14
	The surface severely shrinks.	<8
Smell	It is intensely fruity and sweet.	≥14, <20
	It is lightly fruity.	≥8, <14
	It is not fruity.	<8
Taste	The flesh is crisp, tender, and juicy.	≥14, <20
	The flesh is not crisp and less juicy.	≥8, <14
	The flesh is soft and not juicy.	<8
Acceptability	Fully acceptable.	≥14, <20
	Basically acceptable.	≥8, <14
	Unacceptable.	<8

## Data Availability

Not applicable.

## Supplementary Materials

The following supporting information can be downloaded at: https://www.mdpi.com/article/10.3390/foods11101470/s1, Figure S1: The total bacterial count of fresh-cut apples during storage after treatment with phytic acid or the control. Vertical bars represent standard errors of the means of three replicates. The different letters indicate significant differences at *p* < 0.05; Figure S2: The water loss rate of fresh-cut apples during storage after treatment with phytic acid or the control. Vertical bars represent standard errors of the means of three replicates. The different letters indicate significant differences at *p* < 0.05.

## Abbreviations

4CL—4-coumarate: CoA ligase; ANOVA—one-way analysis of variance; BI—browning index; C4H—cinnamate 4-hydroxylase; CAT—catalase; MDA—malonaldehyde; OD—optical density; PAL—phenylalanine ammonia-lyase; POD—peroxidase; PPO—polyphenol oxidase; ROS—reactive oxygen species; SOD—superoxide dismutase.
