# Supplementary material for: Phytic Acid Treatment Inhibits Browning and Lignification to Promote the Quality of Fresh-Cut Apples during Storage
Source: Foods. 2022 May 18;11(10):1470. doi: 10.3390/foods11101470 (PMC9140707; doi:10.3390/foods11101470)
Supplement: Supplementary file 1 [file foods-11-01470-s001.zip › foods-1694356-supplementary.pdf]

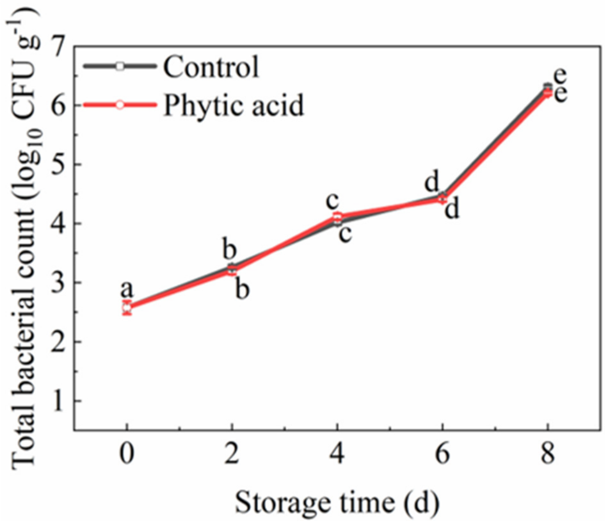

Supplementary Figure S1. The total bacterial count of fresh−cut apples during storage after treatment with phytic acid or the control. Vertical bars represent standard errors of the means of three replicates. The different letters indicate significant differences at $p < 0.05$.

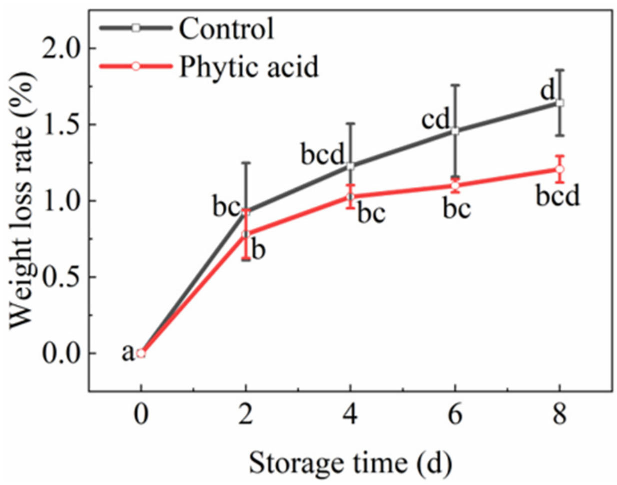

Supplementary Figure S2. The water loss rate of fresh−cut apples during storage after treatment with phytic acid or the control. Vertical bars represent standard errors of the means of three replicates. The different letters indicate significant differences at $p < 0.05$.
